# PF4 regulates neuronal ferroptosis in cerebral hemorrhage through CXCR3/PI3K/AKT/Nrf2 pathway

**DOI:** 10.17305/bb.2024.11415

**Published:** 2024-11-26

**Authors:** Na Hu, Yunfeng Li, Guohong Zhang, Wei Wang, Liping An, Ran An, Yu Liu

**Affiliations:** 1 Department of Biochemistry and Biology, School of Pharmacy, Hebei University of Chinese Medicine, Shijiazhuang, Hebei Province, China; 2 Hebei Key Laboratory of Chinese Medicine Research on Cardio-Cerebrovascular Disease, Shijiazhuang, Hebei Province, China

**Keywords:** Platelet factor 4, PF4, intracerebral hemorrhage, ICH, ferroptosis, C-X-C motif chemokine receptor 3, CXCR3/phosphatidylinositol 3-kinase (PI3K)/AKT/nuclear factor erythroid-2-related factor 2 (Nrf2) pathway

## Abstract

Inhibiting ferroptosis represents a promising strategy for managing neuronal injury caused by intracerebral hemorrhage (ICH). Platelet factor 4 (PF4), a chemokine with diverse biological functions, has an unclear role in ICH and its impact on neuronal ferroptosis. To investigate this, a hemin-induced injury model was established in PC12 cells *in vitro*, and an ICH model was created *in vivo* using IV collagenase injection. Hemin-treated PC12 cells were co-cultured with recombinant mouse PF4 (Rm-PF4) protein to examine the effects of PF4 on ferroptosis. Additionally, Rm-PF4 was administered intraperitoneally to ICH mice, and its influence on neurological dysfunction, brain edema, and neuronal ferroptosis was evaluated. Western blot analysis was employed to assess PF4 levels, CXCR3/phosphatidylinositol 3-kinase (PI3K)/AKT/nuclear factor erythroid-2-related factor 2 (Nrf2) pathway activation, and ferroptosis-related protein expression. PF4 levels were found to be reduced in both perihematomal brain tissues of ICH mice and hemin-treated PC12 cells. Treatment with Rm-PF4 decreased ferrous ion, malondialdehyde (MDA), and reactive oxygen species (ROS) levels, effectively inhibiting ferroptosis in PC12 cells. Furthermore, Rm-PF4 administration alleviated neurological dysfunction, neuronal damage, and brain edema while suppressing neuronal ferroptosis in ICH mice. Mechanistically, Rm-PF4 activated the CXCR3/PI3K/AKT/Nrf2 pathway, and this protective effect was diminished by a CXCR3 antagonist in both ICH mice and hemin-treated PC12 cells. In conclusion, PF4 mitigates ICH-induced neuronal ferroptosis in mouse models and PC12 cells by activating the CXCR3/PI3K/AKT/Nrf2 pathway.

## Introduction

Intracerebral hemorrhage (ICH) accounts for approximately 10% to 15% of all stroke cases but is responsible for a striking 50% of stroke-related deaths [1, 2]. Globally, around 2.8 million people die from ICH annually, with a 30-day mortality rate as high as 50% [3, 4]. Beyond its high mortality, ICH leaves survivors with varying degrees of neurological dysfunction, necessitating long-term rehabilitation and imposing a significant burden on families and society [5]. Brain damage following ICH can be classified into primary and secondary injury. Secondary injury, driven by ferroptosis, inflammation, and oxidative stress, plays a crucial role in determining patient outcomes [4, 6]. Despite ongoing efforts, current interventions for ICH have yielded limited success, underscoring the urgent need to better understand the mechanisms of ICH and identify novel therapeutic targets. In 2012, researchers identified ferroptosis, a unique form of cell death marked by iron accumulation, lipid peroxidation, loss of glutathione peroxidase 4 (GPX4) activity, and elevated production of reactive oxygen species (ROS) [7, 8]. Ferroptosis is regulated by a complex network of signaling molecules and metabolic pathways, and it has been linked to the pathogenesis of various diseases, including nervous system disorders, kidney injury, and cancer [9, 10]. After ICH, excessive iron ions and hemoglobin released from the hematoma contribute to neurotoxicity, accelerate neural degeneration, and promote neuronal ferroptosis [11, 12]. For instance, Cao et al. [13] demonstrated that Liproxstatin-1, a ferroptosis inhibitor, reduced neurological dysfunction, brain edema, and neuroinflammation following subarachnoid hemorrhage, thereby mitigating neuronal cell death. These findings highlight ferroptosis as a critical driver of secondary damage in ICH, suggesting that targeting ferroptosis could provide a promising therapeutic approach for ICH-induced neuronal injury.

Platelet factor 4 (PF4) is a protein synthesized by the alpha granules of platelets. Structurally, it is a tetramer of basic polypeptides consisting of 70 amino acids [14, 15]. PF4 performs a wide range of biological functions, including the inhibition of vascular endothelial cell growth, regulation of platelet aggregation, and antibacterial and antiviral activities. Additionally, it plays roles in the inflammatory response, immune regulation, and angiogenesis [16]. In recent years, researchers have investigated the relationship between PF4, inflammation, and aging. Notably, PF4 has been shown to reduce neuroinflammation, improve cognitive function, and enhance synaptic plasticity in aged mice [17–19]. However, the role of PF4 in ICH and its potential contribution to neuronal ferroptosis remains largely unknown.

The phosphatidylinositol 3-kinase (PI3K)/protein kinase B (AKT) signaling pathway is pivotal for linking extracellular signals to intracellular responses. It plays a key role in processes, such as cell proliferation, apoptosis, and oxidative stress [20]. Additionally, nuclear factor erythroid-2-related factor 2 (Nrf2) is crucial for regulating cellular antioxidant responses by encoding genes involved in antioxidant, anti-inflammatory, and detoxification pathways [21]. Importantly, dysregulation of the PI3K/AKT pathway and Nrf2 activity has been closely associated with neuronal injury following ICH [22, 23].

To address these gaps, we developed a hemin-treated PC12 cell model and an ICH mouse model to investigate the effects of recombinant mouse PF4 (Rm-PF4) on neuronal ferroptosis. We also examined whether PF4 exerts its effects through the PI3K/AKT/Nrf2 pathway. This study aims to elucidate the mechanisms by which PF4 alleviates neuronal damage and to provide a novel therapeutic approach for treating ICH in clinical settings.

## Materials and methods

### Cell culture and ICH model construction *in vitro*

Highly differentiated PC12 cells (SNL-124) were obtained from Sunncell Biotechnology Co., Ltd. (Wuhan, Hubei, China). Prior to experimentation, the cells were cultured in a mixture of DMEM medium (11965092, Gibco, Grand Island, NY, USA) and 10% fetal bovine serum (A5670701, Gibco) at 37 ^∘^C with 5% CO_2_. The medium was changed every three days, and the cells were passaged at a ratio of 1:3. According to Lu et al. [24], when the cells reached 70%–80% confluence, PC12 cells were treated with 80 µM hemin (ST1375, Beyotime, Shanghai, China) for 24 h to establish an *in vitro* ICH model. One hour after the hemin treatment, Rm-PF4 (100 ng/mL, HY-P71885, MedChemExpress, Monmouth Junction, NJ, USA) was introduced, and co-culturing with PC12 cells continued for an additional 23 h, forming the hemin+Rm-PF4 group. In addition, the hemin+Rm-PF4+LY294002 group was created by adding the PI3K/AKT pathway inhibitor LY294002 (25 µM, HY-10108, MedChemExpress) to the hemin+Rm-PF4 group. The hemin+Rm-PF4+ML385 group was treated with the Nrf2 inhibitor ML385 (5 µM, HY-100523, MedChemExpress), while the hemin+Rm-PF4+AMG487 group received the CXCR3 antagonist AMG487 (1 µM, HY-15319, MedChemExpress) in addition to hemin+Rm-PF4.

### Cell morphology observation

To investigate the effect of hemin on PC12 cell death, cells were initially exposed to hemin for 30 min, followed by treatment with various inhibitors for 24 h: ferroptosis inhibitor ferrostatin-1 (Fer-1, 2.5 µM, HY-100579), iron chelator deferoxamine (DFO, 100 µM, HY-B1625), antioxidant N-acetylcysteine (NAC, 10 mM, HY-B0215), autophagy inhibitor 3-methyladenine (3-MA, 10 mM, HY-19312), apoptosis inhibitor Z-VAD-FMK (10 µM, HY-16658B), or necrosis inhibitor necrostatin-1 (Nec-1, 10 µM, HY-15760) [25]. All reagents were purchased from MedChemExpress. The treated PC12 cells were observed using a microscope (DM IL LED, Leica, Heidelberg, Germany), with random fields of view selected for photography.

### Cell counting kit-8 (CCK-8) assay

PC12 cells were seeded into 96-well culture plates at a density of 2.0 × 10^ImEquation2^ cells/well. Once the cells had fully adhered to the plate, the old culture medium was discarded, and fresh medium containing hemin and various inhibitors was added to each well. After 24 h of treatment, 20 µL of CCK-8 reagent (C0038, Beyotime) was added to each well. Following a 2-h incubation at 37 ^∘^C, protected from light, the OD450 values were measured using a microplate reader (1410101, Thermo Fisher Scientific, Waltham, MA, USA) to assess cell viability.

### Flow cytometry

After being rinsed twice with PBS, PC12 cells were gently resuspended in 500 µL of Binding Buffer. Next, 5 µL of propidium iodide (HY-K1073, MedChemExpress) and 5 µL of Annexin-V-FITC were added, and the cells were incubated for 15 min. Samples were then analyzed by flow cytometry using a BD FACSCalibur™ (BD Biosciences, San Jose, CA, USA). Cell mortality was assessed using FlowJo software (v10.8, BD Biosciences).

### Transmission electron microscopy for cellular ultrastructure

PC12 cells were subjected to various treatments, and the precipitate was collected by centrifugation. The samples were then fixed with 2.5% glutaraldehyde (G6257, Sigma-Aldrich, St. Louis, MO, USA) at 4^∘^C overnight. After fixation, the samples were rinsed three times with PBS. Following this, they were fixed with 1% osmium tetroxide (O5500, Sigma-Aldrich) for 2 h.

Dehydration was performed using a gradient of ethanol (30%, 50%, 70%, 80%, 95%, and 100%) for 10 min at each concentration, ending with a final dehydration step using 100% ethanol. The samples were then further dehydrated twice with 100% acetone for 10 min each.

Following dehydration, the samples were embedded and polymerized at 60 ^∘^C for 48 h. After polymerization, the samples were sectioned to a thickness of 60 nm and double-stained with 2% uranyl acetate and lead citrate (YS25690U, YaJi Biological, Shanghai, China). The sections were then dried after being mounted on copper grids.

The samples were observed under a transmission electron microscope (HT7800, HITACHI, Tokyo, Japan), and images were captured for analysis.

### Measurement of ferrous ion levels

PC12 cells were collected and rinsed with serum-free medium. The FerroOrange Fluorescent Probe (HY-D1913, MedChemExpress) was then introduced at a concentration of 1 µM and thoroughly mixed, followed by incubation in the dark for 30 min. After the incubation, the cells were not washed, and fluorescence microscopy was immediately performed to observe the cells. Photographs were taken to document the fluorescence intensity.

### Measurement of cellular ROS levels

After being subjected to various treatments, PC12 cells were rinsed twice with PBS. The cells were then incubated with the DCFH-DA fluorescent probe (10 µM, HY-D0940, MedChemExpress) for 20 min in a light-free environment. Following incubation, the cells were collected by centrifugation, rinsed twice with PBS, and resuspended in serum-free culture medium. The fluorescence intensity of intracellular DCFH-DA was analyzed using flow cytometry to quantify ROS levels.

### *In vivo* ICH model construction

Male C57BL/6 mice, aged 8–10 weeks and weighing 25–30 g, were obtained from Vitalriver (Beijing, China). The mice were housed in a controlled environment at 22 ^∘^C with 55%–60% humidity and a 12-h light-dark cycle. Prior to the experiment, the mice were acclimatized for two weeks in a barrier environment, during which all areas of the animal facility, including cage boxes, food containers, water bottles, and drinking spouts, were regularly cleaned and disinfected.

As described by Duan et al. [26], the mice were rendered unconscious and immobilized by intraperitoneal injection of 2% sodium pentobarbital. Type IV collagenase (C4-BIOC, Sigma-Aldrich) (0.1 U) was dissolved in 1 µL of saline and injected into the right basal ganglia of the mice at a rate of 0.3 µL/min to induce an ICH model. In the sham group (Sham), only 1 µL of saline was injected, with the remaining procedures identical. Once the ICH model was successfully established, Rm-PF4 (100 µg/kg) was administered intraperitoneally to the mice, forming the ICH+Rm-PF4 group. In addition, the ICH+Rm-PF4+Erastin group received an intraperitoneal injection of Erastin (20 mg/kg, HY-15763). The ICH+Rm-PF4+AMG487 group received a subcutaneous injection of AMG487 (5 mg/kg) based on the ICH+Rm-PF4 group. For the ICH+Rm-PF4+LY294002 group, mice were intraperitoneally injected with LY294002 (10 nM/2 µL) [27]. Finally, the ICH+Rm-PF4+ML385 group was treated with intraperitoneal ML385 (30 mg/kg) [28] based on the ICH+Rm-PF4 group. All experiments were performed in accordance with the ethical guidelines for laboratory animal welfare at Hebei University of Chinese Medicine and were approved by the Laboratory Animal Ethics Committee of the university.

### Behavioral tests

In line with the methodology outlined by Liu et al. [29], we assessed the neurological function of mice 24 h after successful ICH modeling using the modified Garcia score and balance beam test. The Garcia score comprises six criteria, each rated on a scale of 0–3, yielding a total score between 3 and 18 points. In the balance beam test, mice were evaluated based on their ability to walk across a narrow wooden beam for one minute, with scores ranging from 0 to 4, where higher scores indicate better neurological function. Additionally, following the approach of Xiao et al. [30], neurological deficits were scored across six criteria: body symmetry, forelimb symmetry, circling behavior, climbing, gait, and forced circling. Each criterion was rated from 0–3, with a cumulative score ranging from 0–18 points. A higher score indicated greater neurological impairment.

### Brain water content detection

Following the completion of behavioral analysis tests, the mice were anesthetized, euthanized, and their brain tissue was obtained by dissection on ice. The wet weight of brain tissue was measured immediately, followed by a 24-h drying period at 100 ^∘^C, and its dry weight was measured. Brain water content (%) ═ (wet weight - dry weight) / wet weight × 100%.

### Nissl staining

Mouse brain tissues were exposed to 4% paraformaldehyde (P0099, Beyotime) for 48 h, sectioned after paraffin embedding (thickness 4–5 µm), and deparaffinized with xylene (247642, Sigma-Aldrich). The sections were then dehydrated through a gradient ethanol series. Following the method outlined by Zhang et al. [31], Nissl staining solution (G1434, Solarbio, Beijing, China) was added dropwise and incubated at 37 ^∘^C for 10 min. The tissues were washed twice with distilled water, then dehydrated with 95% ethanol for 2 min. After treatment with xylene for 5 min, the samples were sealed with neutral gum. Staining was assessed under an inverted microscope to evaluate neuronal damage in the brain tissue.

### Prussian blue staining

Iron levels in brain tissues were evaluated using a Prussian blue staining kit (G1422, Solarbio). Mouse brain tissue sections were deparaffinized with xylene, followed by rehydration through a series of ethanol solutions, and then washed with distilled water. The sections were stained with Prussian blue for 15 min and rinsed thoroughly with distilled water. The nuclei were stained with eosin for 15–30 s, followed by a 30-s rinse in distilled water. After dehydration through an ethanol gradient, the sections were treated with xylene for permeabilization, covered with neutral gum, and examined under a light microscope.

### Measurement of tissue ROS levels

Frozen brain tissue sections from nude mice, collected within 1 h after surgical excision, were covered with drops of cleaning solution and allowed to sit for 5 min. After aspirating the cleaning solution, 10-µM Dihydroethidium (DHE) probe (S0063, Beyotime) was applied dropwise to ensure even coverage of the tissue sections, and the sections were incubated in the dark for 30 min. After two washes with PBS, coverslips were added. The sections were then observed under an inverted microscope, photographed, and analyzed using ImageJ software (version 1.54 h, Wayne Resband, National Institute of Mental Health, USA).

**Figure 1.**
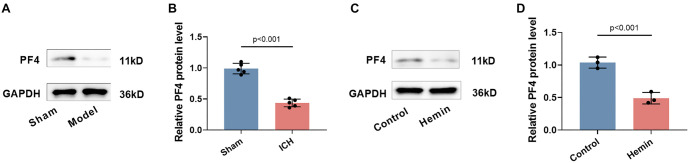
**ICH causes downregulation of PF4 expression.** (A and B) Western blot analysis of PF4 expression in brain tissue from ICH mice. Quantification of PF4 protein levels normalized to GAPDH (*n* ═ 5); (C and D) Western blot showing PF4 protein expression in hemin-treated PC12 cells, with quantification (*n* ═ 3). *P* < 0.05 indicates a statistically significant difference compared to Control groups. ICH: Intracerebral hemorrhage; PF4: Platelet factor 4.

### Iron content test

The iron content in mouse brain tissue was determined using the tissue iron content assay kit (BC4355, Solarbio). Approximately 0.1 g of brain tissue was weighed and homogenized with 1 mL of extraction solution in an ice bath. After centrifugation, the supernatant was collected. Reagents I and II were then added according to the kit instructions, mixed thoroughly, and incubated for 5 min. Chloroform was added, and the mixture was shaken well. After centrifugation, the supernatant was aspirated, and the absorbance was measured at 520 nm to determine the iron content.

### Superoxide dismutase (SOD), 4-HNE and malondialdehyde (MDA) levels test

PC12 cells or mouse brain tissue were lysed by ultrasonication at 4 ^∘^C. The lysate was then centrifuged, and the resulting supernatant was collected. The levels of 4-HNE, SOD, and MDA in the PC12 cells or brain tissues were determined using the Lipid Peroxidation (4-HNE) Assay Kit (ab238538, Abcam, Cambridge, MA, USA), the SOD Detection Kit (S0101S, Beyotime), and the MDA Detection Kit (S0131S, Beyotime), following the manufacturer’s instructions.

### Western blot

RIPA lysis buffer (P0013B, Beyotime) was used to lyse cells or tissues for protein extraction, and protein concentrations were determined using the BCA kit (P0012, Beyotime). The extracted proteins were separated by electrophoresis and then transferred to a PVDF membrane (88518, Invitrogen). The membrane was subsequently blocked with 5% bovine serum albumin (V900933, Sigma-Aldrich) for 2 h. After rinsing the membrane, it was incubated overnight at 4 ^∘^C with the following primary antibodies: PF4 (ab9561, 1:1000, Abcam), Glutathione peroxidase 4 (GPX4, ab41787, 1:100, Abcam), acyl-CoA synthetase long-chain family member 4 (ACSL4, PA5-27137, 1:500, Invitrogen), Solute carrier family 7 member 11 (SLC7A11, PA1-16893, 1:1000, Invitrogen), Cyclooxygenase-2 (COX-2, 35–8200, 1:100, Invitrogen), p-PI3K (PA5-104853, 1:1000, Invitrogen), PI3K (MA1-74183, 1:1000, Invitrogen), Akt (44-609G, 1:1000, Invitrogen), p-Akt (44-621G, 1:1000, Invitrogen), Nrf2 (ab137550, 1:2000, Abcam), Heme Oxygenase-1 (HO-1, MA1-112, 1:1000, Invitrogen), NAD(P)H: Quinone Oxidoreductase 1 (NQO1, ab97385, 1:1000, Abcam), and CXCR3 (702228, 1:100, Invitrogen). The next day, the membranes were rinsed three times and then incubated with HRP-labeled goat anti-rabbit IgG (31460, 1:10000, Invitrogen) for 2 h. Afterward, the membranes were treated with ECL chemiluminescent substrate (34580, Thermo Fisher Scientific) and visualized using a gel imaging system (iBright CL1500, Invitrogen). The grayscale values of each protein band were quantified using ImageJ software, with protein levels normalized to GAPDH (MA1-16757, 1:1000, Invitrogen).

### Ethical statement

The animal experiment protocol received approval from the Experimental Animal Ethics Committee of Hebei University of Chinese Medicine (DWLL202402091).

### Statistical analysis

A minimum of three repetitions were performed in each experiment, with results reported as mean ± standard deviation. For statistical analysis and image plotting, we used SPSS 26.0 (IBM SPSS Statistics 26) and Prism software (GraphPad 9.0). A normality test and homogeneity of variance test were conducted to confirm that the data followed a normal distribution and had homogeneous variance. Subsequently, Student’s *t*-test and analysis of variance (ANOVA) were performed to compare different groups. A *P* value of *P* < 0.05 indicates a statistically significant difference.

## Results

### ICH leads to downregulation of PF4 expression

The levels of PF4 expression in brain tissue were assessed using Western blot analysis. The results showed a significant decrease in PF4 protein expression in the brain tissue following ICH (*P* < 0.001, Figure 1A and 1B). Additionally, PF4 expression was significantly reduced in PC12 cells following hemin treatment (*P* < 0.001, Figure 1C and 1D), suggesting that ICH leads to downregulation of PF4 expression.

### Hemin induces ferroptosis in PC12 cells

**Figure 2.**
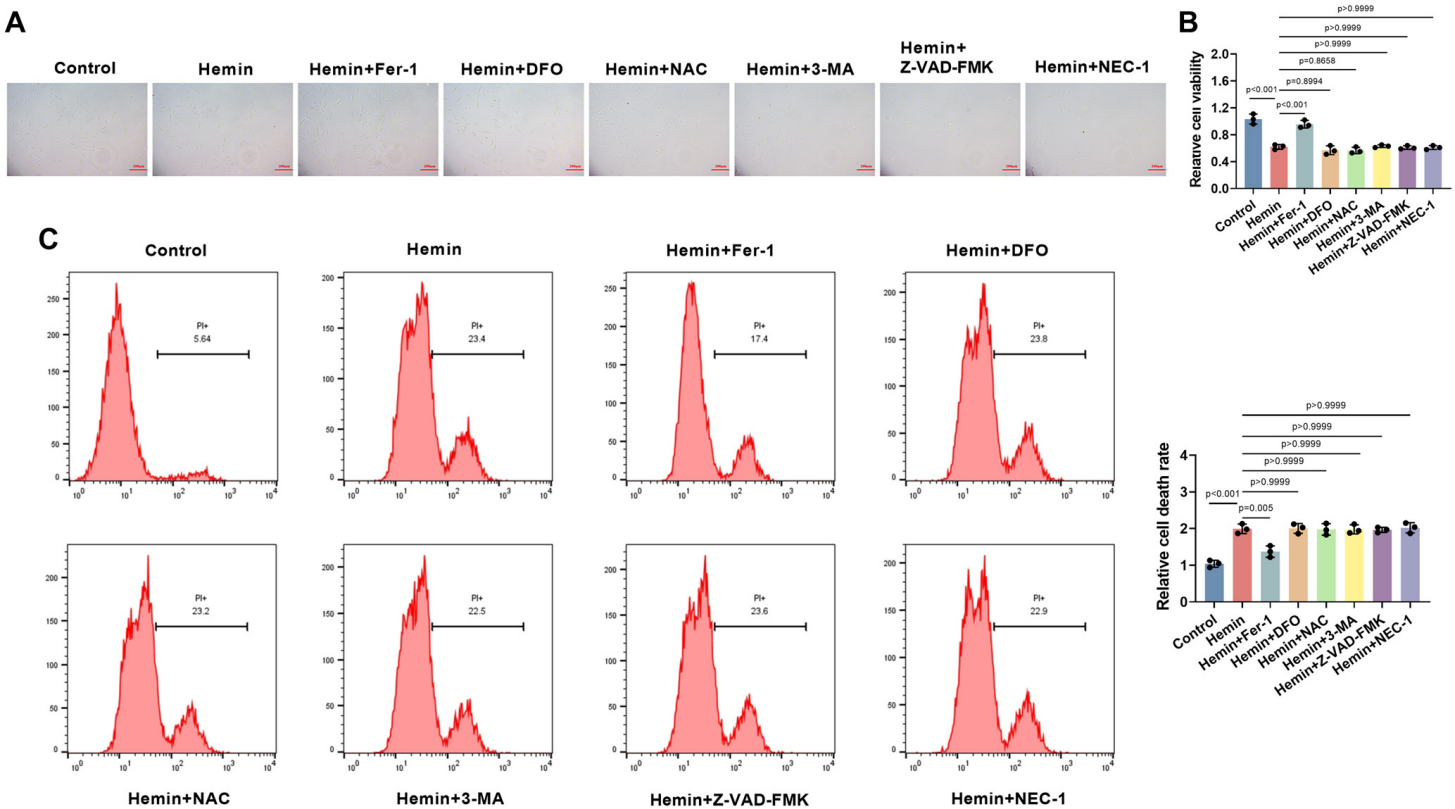
**Hemin can induce ferroptosis in PC12 cells.** (A) Light microscopy images of PC12 cells treated with hemin (80 µM), alone or in combination with Fer-1 (ferroptosis inhibitor), DFO (iron chelator), NAC (antioxidant), 3-MA (autophagy inhibitor), Z-VAD-FMK (apoptosis inhibitor), or Nec-1 (necrosis inhibitor) for 24 h (10×, bar ═ 200 µm); (B) Cell viability assessed using the CCK-8 assay after treatments (*n* ═ 3); (C) Flow cytometry analysis of cell death in PC12 cells after treatment, quantified as percentage of total cells. *P <* 0.05 vs. control. CCK-8: Cell counting kit-8.

To investigate the impact of hemin on PC12 cells, various inhibitors were added to the cell culture system: Fer-1 (ferroptosis inhibitor), DFO (iron chelator), NAC (antioxidant), 3-MA (autophagy inhibitor), Z-VAD-FMK (apoptosis inhibitor), and NEC-1 (necrosis inhibitor). In the control group, cells displayed a typical, spiky morphology with multiple protrusions, and they were numerous with intact cellular structure. In contrast, hemin-treated PC12 cells showed a marked reduction in number and a significant decrease in cell viability (*P* < 0.001). The addition of Fer-1 alleviated the effects of hemin, significantly improving cell viability (*P* < 0.001), whereas other inhibitors did not produce a significant effect, suggesting that hemin-induced cell death is primarily mediated by ferroptosis (Figure 2A and 2B). Flow cytometry analysis further revealed a significant increase in PC12 cell death after hemin treatment (*P* < 0.001), and the addition of Fer-1 significantly reduced cell mortality (*P* ═ 0.005). In contrast, other inhibitors had no significant effect, further supporting the conclusion that hemin induces ferroptosis in PC12 cells (Figure 2C).

### Rm-PF4 blocks ferroptosis induced by hemin in PC12 cells

**Figure 3.**
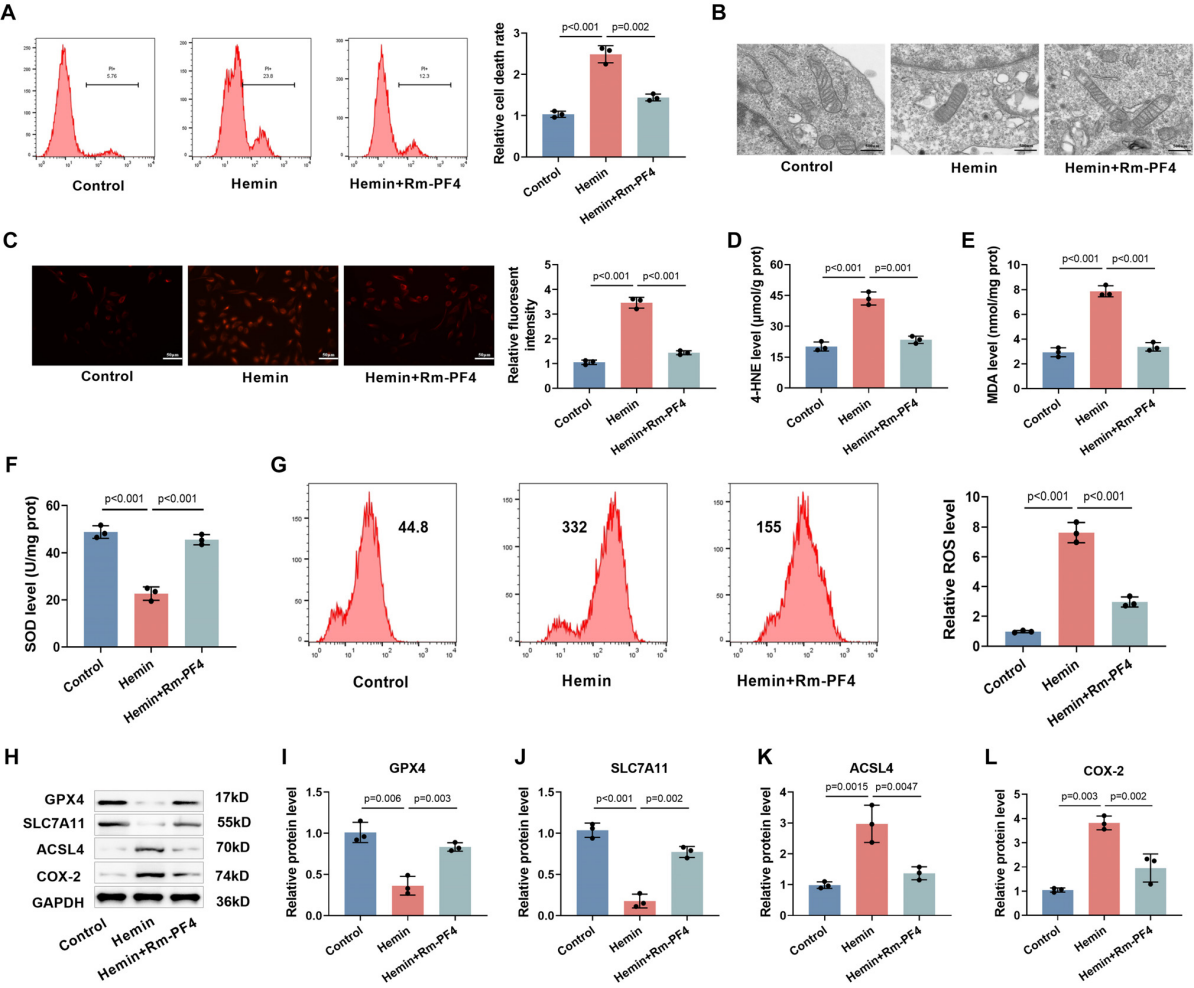
**Rm-PF4 inhibited hemin-induced ferroptosis of PC12 cells.** (A) Flow cytometry analysis of PC12 cell mortality after treatment with hemin and Rm-PF4 (100 ng/mL), quantified as percentage of total cells; (B) Transmission electron microscopy (TEM) images showing mitochondrial morphology in PC12 cells after hemin treatment with or without Rm-PF4 (20k×, bar ═ 500 nm); (C) FerroOrange staining for detection of intracellular ferrous ion levels in PC12 cells (40×, bar ═ 50 µm); (D–F) Measurement of MDA, 4-HNE, and SOD levels in PC12 cells using specific assay kits; (G) Detection of intracellular ROS in PC12 cells using DCFH-DA fluorescent probe; (H–L) Western blot analysis of ferroptosis-related proteins (GPX4, SLC7A11, ACSL4, and COX-2) in PC12 cells treated with hemin and Rm-PF4. Data are shown as mean ± SD (*n* ═ 3), with *P* < 0.05 indicating statistical significance compared to the Control group. ROS: Reactive oxygen species; SOD: Superoxide dismutase; MDA: Malondialdehyde; Rm-PF4: Recombinant mouse PF4; GPX4: Glutathione peroxidase 4.

After 1 h of hemin intervention, PC12 cells were co-cultured with Rm-PF4 to investigate the effect of PF4 on ferroptosis induced by hemin. The cell mortality rate was significantly increased after hemin treatment (*P* < 0.001), whereas the addition of Rm-PF4 significantly reduced the cell death rate (Figure 3A). The mitochondrial morphology of PC12 cells was observed using transmission electron microscopy. The results showed that, after hemin treatment, numerous lipid vacuoles were visible in the cytoplasm, the number of mitochondria was notably reduced, and the mitochondrial cristae were disrupted or even disappeared. In contrast, Rm-PF4 treatment reversed these mitochondrial changes induced by hemin (Figure 3B). Using the FerroOrange ferrous ion fluorescence probe, we observed a significant increase in the fluorescence intensity of ferrous ions in PC12 cells following hemin treatment (*P* < 0.001). Rm-PF4 treatment attenuated this effect (Figure 3C).

Additionally, hemin treatment caused significant increases in 4-HNE and MDA levels (Figure 3D and 3E), a marked decrease in SOD levels (Figure 3F), and a notable increase in ROS levels (Figure 3G) in PC12 cells (*P* < 0.001). Rm-PF4 treatment rescued these hemin-induced changes. Furthermore, after hemin treatment, the protein levels of GPX4 and SLC7A11 were significantly reduced, while the levels of ACSL4 and COX-2 were notably increased. The addition of Rm-PF4 attenuated these alterations in protein expression (Figure 3H–3L). These findings suggest that hemin triggers ferroptosis in PC12 cells, while Rm-PF4 prevents hemin-induced ferroptosis.

### Rm-PF4 inhibits hemin-induced ferroptosis in PC12 cells via activating the PI3K/AKT/Nrf2 pathway

**Figure 4.**
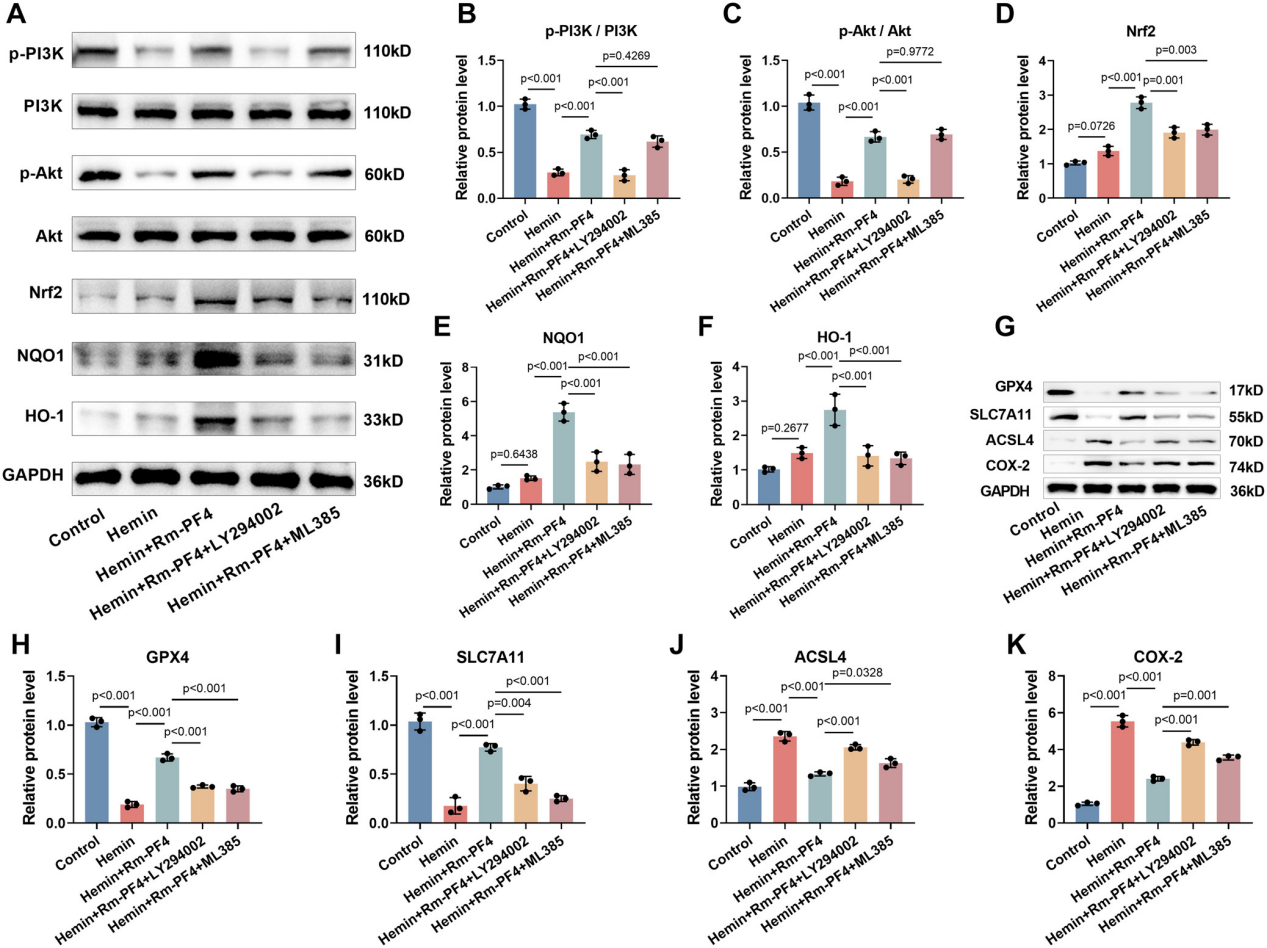
**Rm-PF4 inhibits hemin-induced ferroptosis in PC12 cells by activating PI3K/AKT/Nrf2 pathway.** (A–F) Western blot analysis of PI3K/AKT/Nrf2 pathway proteins (p-PI3K, PI3K, p-AKT, AKT, Nrf2, HO-1, and NQO1) in PC12 cells treated with hemin and Rm-PF4, with or without PI3K inhibitor (LY294002) or Nrf2 inhibitor (ML385); (G–K) Western blot showing the effect of pathway inhibition on ferroptosis-related protein levels (GPX4, SLC7A11, ACSL4, and COX-2) in PC12 cells. Data are shown as mean ± SD (*n* ═ 3), with *P <* 0.05 vs control, ns: Non-significant. Rm-PF4: Recombinant mouse PF4; GPX4: Glutathione peroxidase 4; PI3K: Phosphatidylinositol 3-kinase; Nrf2: Nuclear factor erythroid-2-related factor 2.

To further explore the molecular mechanisms behind PF4’s ability to reduce ferroptosis, we analyzed the signaling pathways associated with this process. In PC12 cells, hemin treatment led to a significant reduction in the phosphorylation levels of PI3K and Akt, while Nrf2, HO-1, and NQO1 protein levels were mildly elevated, although the variance was not statistically significant. In contrast, treatment with Rm-PF4 resulted in a marked increase in the phosphorylation of PI3K and Akt, as well as elevated levels of Nrf2, HO-1, and NQO1 (Figure 4A–4F). Notably, the PI3K/AKT pathway inhibitor LY294002 caused a substantial decrease in the phosphorylation of PI3K and Akt, as well as a reduction in Nrf2, HO-1, and NQO1 levels. Conversely, the Nrf2 inhibitor ML385 reduced the levels of Nrf2, NQO1, and HO-1, but had little effect on the phosphorylation of PI3K and Akt, suggesting that activation of the PI3K/AKT pathway precedes Nrf2 activation. Additionally, Rm-PF4 attenuated the hemin-induced decrease in GPX4 and SLC7A11 levels and the increase in ACSL4 and COX-2 levels (*P* < 0.001), LY294002 and ML385 weakened the protective effect of Rm-PF4, leading to a notable decline in GPX4 and SLC7A11 levels and a marked increase in ACSL4 and COX-2 levels (Figure 4G–4K). These findings suggest that Rm-PF4 can suppress ferroptosis induced by hemin in PC12 cells through the PI3K/AKT/Nrf2 signaling pathway.

### Rm-PF4 inhibits hemin-induced ferroptosis by mediating PI3K/AKT/Nrf2 pathway activation through CXCR3

**Figure 5.**
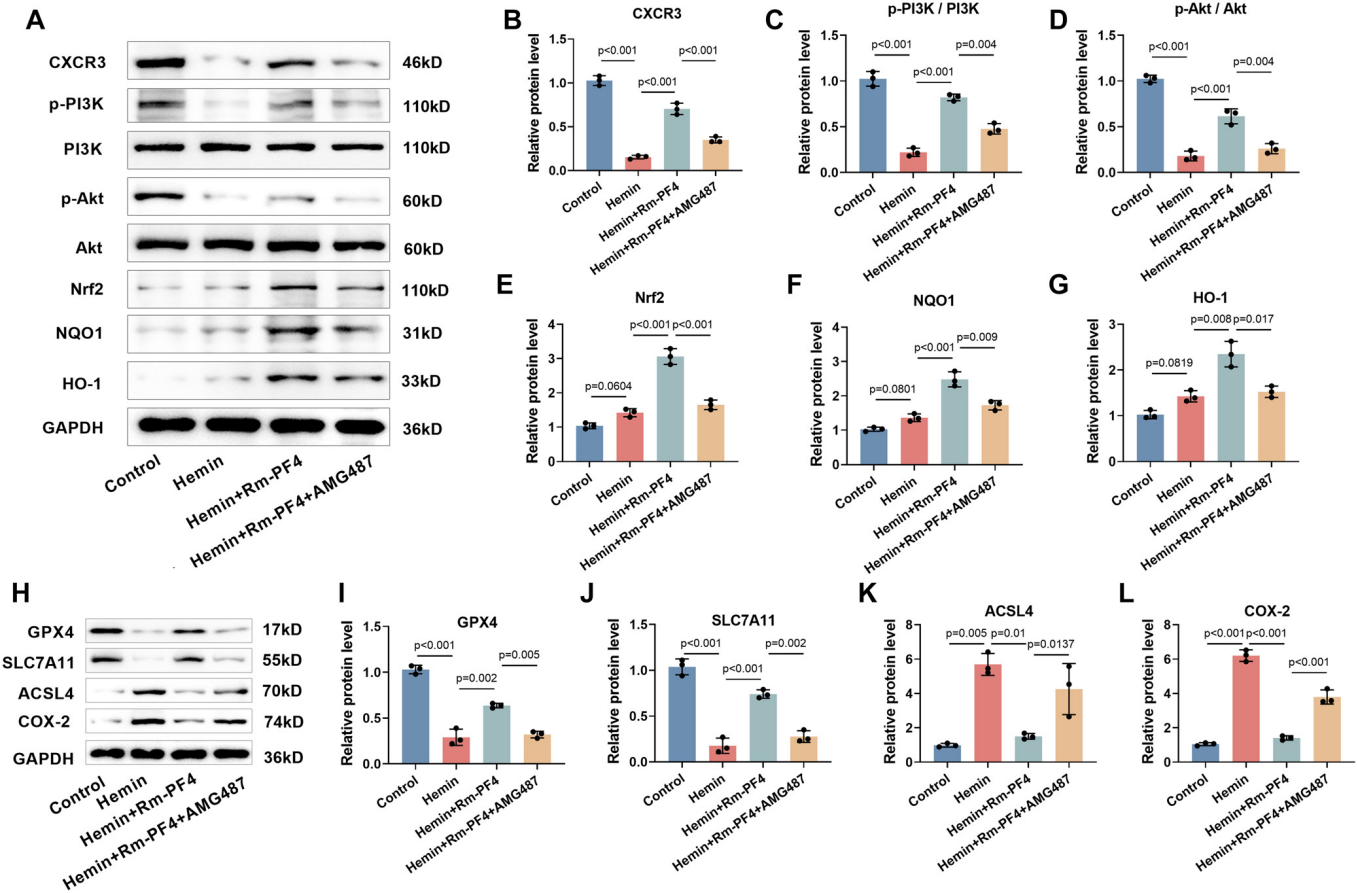
**Rm-PF4 inhibits ferroptosis in PC12 cells induced by hemin via mediating the activation of PI3K/AKT/Nrf2 pathway through CXCR3.** (A–G) Western blot analysis of CXCR3 and PI3K/AKT/Nrf2 pathway proteins in PC12 cells treated with hemin and Rm-PF4, with or without CXCR3 antagonist (AMG487); (H–L) Western blot showing changes in GPX4, SLC7A11, ACSL4, and COX-2 protein levels after CXCR3 inhibition by AMG487. Data are presented as mean ± SD (*n* ═ 3), *P* < 0.05 vs control, ns: Non-significant. Rm-PF4: Recombinant mouse PF4; GPX4: Glutathione peroxidase 4; PI3K: Phosphatidylinositol 3-kinase; Nrf2: Nuclear factor erythroid-2-related factor 2.

Studies have shown that CXCR3 acts as a receptor for PF4 and plays a significant role in brain inflammation in mice [32]. Based on this, we hypothesized that Rm-PF4 could stimulate the PI3K/AKT/Nrf2 pathway by binding to CXCR3. Hemin administration resulted in a reduction in CXCR3 expression in PC12 cells (*P* < 0.001), whereas Rm-PF4 increased CXCR3 levels. However, the CXCR3 antagonist AMG487 reversed this effect (Figure 5A and 5B). Moreover, the influence of Rm-PF4 on PI3K and Akt phosphorylation, as well as Nrf2, NQO1, and HO-1 levels, was diminished by AMG487, suggesting that Rm-PF4 activates the PI3K/AKT/Nrf2 pathway through CXCR3 (Figure 5C–5G). Additionally, AMG487 attenuated the upregulation of GPX4 and SLC7A11, and the downregulation of ACSL4 and COX-2 induced by Rm-PF4 (Figure 5H–5L). These findings indicate that Rm-PF4 mediates PI3K/AKT/Nrf2 pathway activation through CXCR3, thereby alleviating hemin-induced ferroptosis in PC12 cells.

### Rm-PF4 can inhibit neuronal ferroptosis in ICH mice

**Figure 6.**
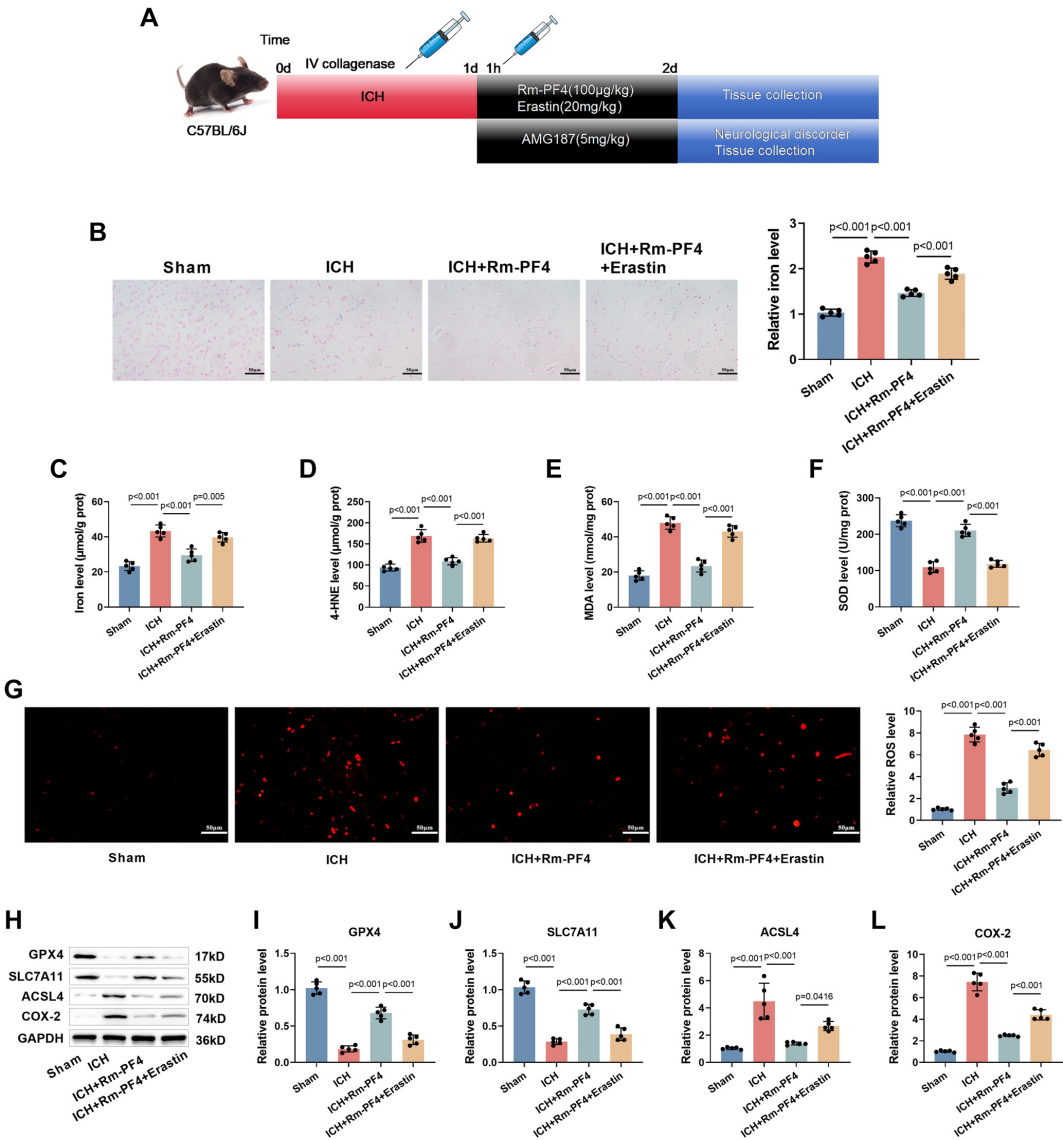
**Rm-PF4 can inhibit neuronal ferroptosis in ICH mice.** (A) Flowchart illustrating the experimental timeline of the ICH mouse model and Rm-PF4 treatment; (B) Prussian blue staining for iron deposition in the cerebral cortex of ICH mice, with quantification of staining intensity (40×, bar ═ 50 µm); (C–F) Measurement of 4-HNE, MDA, and SOD levels in the brain tissue of ICH mice using specific assay kits; (G) Detection of ROS in brain tissue using DCFH-DA staining (red fluorescence) and fluorescence microscopy (40×, bar ═ 50 µm); (H–L) Western blot analysis of ferroptosis-related proteins (GPX4, SLC7A11, ACSL4, COX-2) in brain tissues of ICH mice treated with Rm-PF4. Data are presented as mean ± SD (*n* ═ 5), with *P* < 0.05 vs Sham. ICH: Intracerebral hemorrhage; Rm-PF4: Recombinant mouse PF4; GPX4: Glutathione peroxidase 4; ROS: Reactive oxygen species; SOD: Superoxide dismutase; MDA: Malondialdehyde.

An ICH mouse model was constructed as shown in Figure 6A. Rm-PF4 and/or Erastin were injected intraperitoneally into the mice to investigate the impact of Rm-PF4 on neuronal ferroptosis in ICH. Prussian blue staining revealed significant iron deposition in the brain tissue of ICH mice. This iron deposition was markedly reduced following Rm-PF4 injection, while Erastin counteracted the effect of Rm-PF4 (*P* < 0.001, Figure 6B). Furthermore, iron content, 4-HNE levels, and MDA levels were significantly elevated (Figure 6C–6E), and SOD levels were significantly reduced (Figure 6F) in brain tissue surrounding the hematoma of ICH mice (*P* < 0.001). Injection of Rm-PF4 alleviated these changes, while Erastin diminished the protective effect of Rm-PF4. DHE fluorescence staining demonstrated that Rm-PF4 alleviated the ICH-induced increase in ROS levels (*P* < 0.001), but Erastin attenuated this effect (Figure 6G). Additionally, the levels of GPX4 and SLC7A11 in the brain tissue around the hematoma were significantly reduced, while the levels of ACSL4 and COX-2 were significantly increased (*P* < 0.001). Rm-PF4 mitigated these abnormal expressions, whereas Erastin reduced the impact of Rm-PF4 (Figure 6H–6L). These findings suggest that ICH induces neuronal ferroptosis in mice, and that Rm-PF4 inhibits this process.

### Rm-PF4 inhibits neuronal ferroptosis through CXCR3/PI3K/AKT/Nrf2 pathway in ICH mice

**Figure 7.**
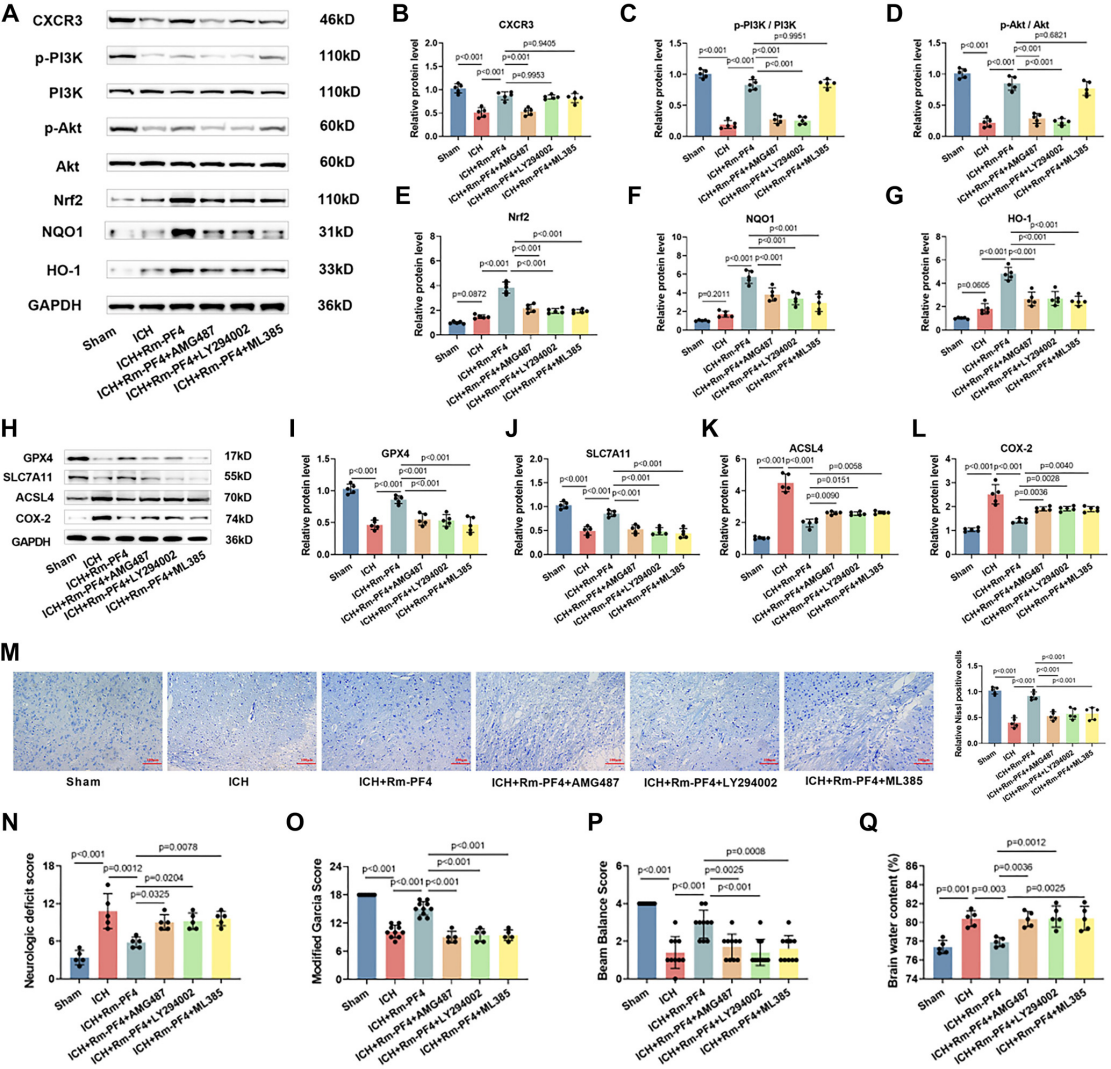
**Rm-PF4 inhibits ferroptosis in ICH mouse neurons via the CXCR3/PI3K/AKT/Nrf2 pathway.** (A–G) Western blot analysis of CXCR3 and PI3K/AKT/Nrf2 pathway proteins (p-PI3K, PI3K, p-AKT, AKT, Nrf2, HO-1, and NQO1) in brain tissues; (H–L) Western blot showing the effect of pathway inhibition on ferroptosis-related protein levels (GPX4, SLC7A11, ACSL4, and COX-2) in brain tissues; (M) Detection of neuronal damage in the cerebral cortex using nissl staining (20×, bar ═ 100 µm); (N) Neurological deficit score (0–18 on a total scale, a higher score correlates with worse neurological function); (O) Improved Garcia score (3∼18 on a total scale, a higher score correlates with less nerve damage); (P) Balance beam score (0∼4 on a total scale, a higher score correlates with less nerve damage); (Q) Measurement of brain water. Data are presented as mean ± SD (*n* ═ 5), with *P* < 0.05 vs Sham, ns: Non-significant. ICH: Intracerebral hemorrhage; Rm-PF4: Recombinant mouse PF4; PI3K: Phosphatidylinositol 3-kinase; Nrf2: Nuclear factor erythroid-2-related factor 2.

Finally, we used the ICH model to investigate whether Rm-PF4 inhibited ferroptosis in mouse neurons through the CXCR3/PI3K/AKT/Nrf2 pathway. We observed a decline in CXCR3 expression, accompanied by a significant reduction in PI3K and Akt phosphorylation levels, with no significant changes in the levels of Nrf2, NQO1, and HO-1 in the perihematoma brain tissue of ICH mice.Injection of Rm-PF4 led to a marked increase in the phosphorylation of PI3K and Akt, as well as elevated levels of CXCR3, Nrf2, NQO1, and HO-1. However, administration of the CXCR3 antagonist AMG487 diminished the effects of Rm-PF4. In addition, the injection of both LY294002 and ML385 increased CXCR3 expression, with LY294002 significantly reducing the phosphorylation levels of PI3K and Akt, as well as the expression levels of Nrf2, NQO1, and HO-1. In contrast, ML385, the Nrf2 inhibitor, only reduced the expression levels of Nrf2, NQO1, and HO-1, with no significant effect on the phosphorylation of PI3K and Akt, which was consistent with the results of our cellular experiments (Figure 7A–7G).

Rm-PF4 also increased the levels of GPX4 and SLC7A11 in brain tissue from ICH mice (*P* < 0.001, Figure 7H–7J), while decreasing the levels of ACSL4 and COX-2 (*P* < 0.001, Figure 7K and 7L). The effects of Rm-PF4 were attenuated by AMG487, LY294002, and ML385. Furthermore, Rm-PF4 improved neuronal damage in ICH mice and increased the number of Nissl-positive cells, but this effect was diminished by AMG487, LY294002, and ML385 (Figure 7M). ICH mice exhibited markedly higher neurologic deficit scores (Figure 7N), significantly lower modified Garcia scores and balance beam scores (Figures 7O and 7P), and higher brain water content (Figure 7Q). While Rm-PF4 injection improved neurological function and reduced brain water contentin ICH mice, the effects were attenuated by AMG487, LY294002, and ML385. These findings suggest that Rm-PF4 inhibits ferroptosis through the CXCR3/PI3K/AKT/Nrf2 pathway, thereby alleviating neuronal injury induced by ICH in mice.

## Discussion

ICH results in a significant number of deaths and disabilities, with nearly two-thirds of survivors suffering from neurological dysfunction, which severely impacts public health [33, 34]. Therefore, exploring the pathogenesis of ICH and mitigating the neuronal damage it causes are crucial for improving the prognosis of ICH patients. The classical experimental model using hemin-treated PC12 cells effectively replicates the process of neuronal injury in ICH, while IV collagenase injection is a common method for inducing ICH in mice [26, 35]. Research indicates that PF4 is highly expressed in the platelets of young mice but less so in older mice, and exogenous PF4 reduces neuroinflammation in the aged hippocampus, suggesting that high PF4 expression is beneficial to the nervous system [18]. Based on this, we hypothesized that elevated PF4 levels might protect against neuronal damage caused by ICH. Our findings showed that PF4 was significantly downregulated in the perihematomal regions of ICH mice and in PC12 cells treated with hemin, suggesting that PF4 may play a role in regulating ICH development. To further investigate its potential protective role, we introduced exogenous PF4 into both the ICH cell model and the ICH mouse model.

Recent studies have revealed a strong correlation between ferroptosis and neuronal damage resulting from ICH [36, 37]. ICH causes the lysis of erythrocytes and the subsequent release of hemin, which is broken down by heme oxygenase-1 (HO-1) into carbon monoxide, iron, and biliverdin [38]. The excess iron then participates in the Fenton reaction, generating ROS and free radicals, which lead to oxidative damage of lipids, proteins, and DNA. This results in a reduction of SOD and GPX4 activities [39]. SLC7A11, a cystine/glutamate countertransporter protein, is critical for regulating ferroptosis and preventing its occurrence [40]. ACSL4 plays a key role in cellular lipid peroxidation and the promotion of ferroptosis, making it one of the signature proteins of this process [41]. COX-2 is crucial for the biosynthesis of ferroptosis-sensitive phospholipids and can catalyze lipid peroxidation in conjunction with ACSL4 [42]. In this study, the ferroptosis inhibitor Fer-1 reduced the inhibitory effect of hemin on PC12 cell viability and diminished cell death, while other inhibitors had no significant impact. Moreover, our findings confirmed that ICH triggered ferroptosis in both mouse neurons and PC12 cells, which is consistent with previous studies [26]. Notably, exogenous PF4 attenuated ferroptosis in mouse neurons and PC12 cells, while Erastin abolished the protective effect of Rm-PF4. This suggests that PF4 may exert its neuroprotective effects by inhibiting ferroptosis.

Several studies have demonstrated that activation of the PI3K/AKT signaling pathway alleviates ferroptosis in various diseases, including traumatic brain injury and cerebral ischemic injury [43, 44]. Nrf2, a downstream gene of the PI3K/AKT pathway, plays a key role in activating HO-1 and NQO1, which are critical in iron/heme metabolism, lipid metabolism, and other metabolic processes [45, 46]. It has also been shown that GPX4 is one of the target genes of Nrf2, and that the Nrf2/GPX4 pathway regulates ferroptosis [47, 48]. In our study, we observed a slight increase in Nrf2 and HO-1 levels in both ICH mice and hemin-treated PC12 cells, although the difference was not statistically significant. This may be due to the maintenance of REDOX balance through cellular regulatory mechanisms, similar to findings reported by Zhou et al. [49]. Additionally, we found a decrease in the phosphorylation levels of PI3K and Akt in both ICH brain tissues and hemin-treated PC12 cells. The activation of the PI3K/AKT pathway occurred before Nrf2 activation, consistent with the findings of Zhang et al. [46]. Notably, the exogenous addition of PF4 activated the PI3K/Akt/Nrf2 pathway and elevated the expression of Nrf2, NQO1, and HO-1. This suggests that PF4 may mitigate ferroptosis by activating the PI3K/Akt/Nrf2 pathway. CXCR3, a chemokine receptor and one of the target proteins of PF4, is critical for cell growth, differentiation, and survival. It is implicated in numerous diseases, including cancer, chronic inflammation, and immune dysfunction [50, 51]. Previous research has shown that PF4 can alleviate age-related neuroinflammation and improve cognitive function in aged mice by binding to CXCR3 [18]. In our study, PF4 increased CXCR3 levels, and the use of CXCR3 antagonists reduced the inhibitory effects of Rm-PF4 on ferroptosis and neurological dysfunction in mice. These results suggest that PF4 may activate the PI3K/AKT/Nrf2 pathway through CXCR3, thereby inhibiting ferroptosis and reducing neuronal damage in ICH-induced mice.

## Conclusion

PF4 mediates the activation of the PI3K/Akt/Nrf2 pathway through CXCR3, which hinders hemin-induced ferroptosis in PC12 cells and alleviates ICH-induced neurological dysfunction, brain edema, and neuronal ferroptosis in mice. This study elucidates the potential mechanism by which PF4 alleviates ICH-induced ferroptosis, offering a new reference for its possible clinical application in managing ICH. However, there are still some limitations, and other protective mechanisms of PF4 against ICH-induced neuronal damage need to be explored further. In this study, PC12 cells were used as a neuronal model. While they are useful for studying neuronal properties, PC12 cells are derived from rat pheochromocytoma and may not fully replicate the behavior of primary neurons. Future studies should use primary neurons to validate these findings. Additionally, the long-term impact of PF4 on ICH outcomes warrants further evaluation. Lastly, whether PF4’s protective effect against ICH-induced neuronal damage is linked to the Nrf2/GPX4 pathway remains to be determined.

## Supplemental data

**Highlights:**

1. The levels of PF4 were decreased both in ICH mice and hemin-treated PC12 cells.

2. PF4 alleviated neuronal damage in ICH mice, alleviated brain edema, and inhibited neuronal ferroptosis.

3. PF4 hindered hemin-induced ferroptosis in PC12 cells.

4. PF4 inhibited ferroptosis by mediating the PI3K/AKT/Nrf2 pathway activation through CXCR3.

**Graphical abstract**


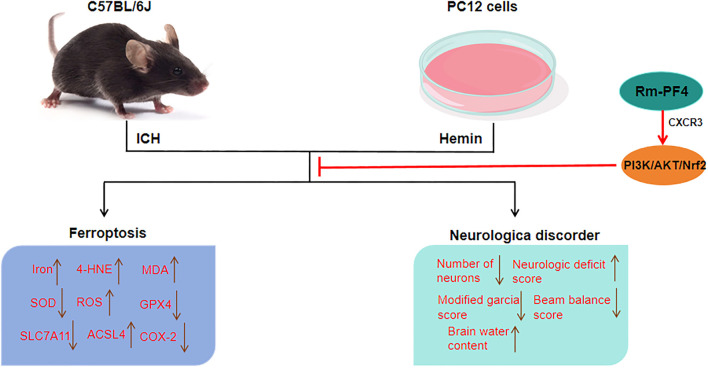


PF4 inhibits hemin-induced ferroptosis in PC12 cells, and weakens ICH-induced neurological dysfunction, brain edema, and neuronal ferroptosis in mice through CXCR3-mediated PI3K/Akt/Nrf2 pathway activation.

## Data Availability

To acquire the data that underpins the results of this study, please contact the corresponding author [YL].
